# Divergent Responses of the Diazotrophic Microbiome to Elevated CO_2_ in Two Rice Cultivars

**DOI:** 10.3389/fmicb.2018.01139

**Published:** 2018-06-01

**Authors:** Yongjie Yu, Jianwei Zhang, Evangelos Petropoulos, Marcos Q. Baluja, Chunwu Zhu, Jianguo Zhu, Xiangui Lin, Youzhi Feng

**Affiliations:** ^1^College of Applied Meteorology, Nanjing University of Information Science and Technology, Nanjing, China; ^2^State Key Laboratory of Soil and Sustainable Agriculture, Institute of Soil Science, Chinese Academy of Sciences, Nanjing, China; ^3^School of Engineering, Newcastle University, Newcastle upon Tyne, United Kingdom

**Keywords:** elevated CO_2_, *nifH*, soil diazotrophs, community structure, co-occurrence network

## Abstract

The species-specific responses of plant growth to elevated atmospheric CO_2_ concentration (eCO_2_) could lead to N limitation and potentially influence the sustainability of ecosystem. Questions remain unanswered with regards to the response of soil N_2_-fixing community to eCO_2_ when developing high-yielding agroecosystem to dampen the future rate of increase in CO_2_ levels and associated climate warming. This study demonstrates the divergent eCO_2_ influences on the paddy diazotrophic community between weak- and strong-responsive rice cultivars. In response to eCO_2_, the diazotrophic abundance increased more for the strong-responsive cultivar treatments than for the weak-responsive ones. Only the strong-responsive cultivars decreased the alpha diversity and separated the composition of diazotrophic communities in response to eCO_2_. The topological indices of the ecological networks further highlighted the different co-occurrence patterns of the diazotrophic microbiome in rice cultivars under eCO_2_. Strong-responsive cultivars destabilized the diazotrophic community by complicating and centralizing the co-occurrence network as well as by shifting the hub species from *Bradyrhizobium* to *Dechloromonas* in response to eCO_2_. On the contrary, the network pattern of the weak-responsive cultivars was simplified and decentralized in response to eCO_2_, with the hub species shifting from *Halorhodospira* under aCO_2_ to *Sideroxydans* under eCO_2_. Collectively, the above information indicates that the strong-responsive cultivars could potentially undermine the belowground ecosystem from the diazotrophs perspective in response to eCO_2_. This information highlights that more attention should be paid to the stability of the belowground ecosystem when developing agricultural strategies to adapt prospective climatic scenarios by growing high-yielding crop cultivars under eCO_2_.

## Introduction

Elevated concentration of atmospheric CO_2_ (eCO_2_) promotes photosynthesis, in turn increases biomass and yield for crop species (Smith et al., 2000; Reich et al., 2001; Ainsworth and Long, 2005). It is found that differences among plant cultivars in their response to eCO_2_ can variously influence their aboveground plant growth as well as the underground nutrient cycling (Bardgett and van der Putten, 2014; García-Palacios et al., 2015; Zhu et al., 2016). For example, it is claimed that rice yield of *Indica* varieties is more positively responsive to eCO_2_ than *Japonica* varieties, with higher crop yield (Hasegawa et al., 2013; Zhu et al., 2015). Based on this unique characteristic, selection and breeding of strong responsive rice cultivars could be a promising strategy to increase crop yield and assure food safety under future climate regimes. It is also demonstrated that belowground N limitation under eCO_2_ constrains sustainability of ecosystem (Reich et al., 2006). Zhu et al. (2015) demonstrated that soil N uptake significantly increased for high-yielding rice cultivars under eCO_2_. Thus, investigation on the sustainability of agroecosystem when building up strong-responsive rice cultivars under eCO_2_ requires further exploration. Soil microorganisms are one of the most indispensable constituents of ecosystem that could greatly contribute to agroecosystem sustainability. Emerging evidence show that differences among plant cultivars in their response to eCO_2_ can influence the soil microbiome and potentially alter the sustainability of soil fertility (Berendsen et al., 2012; Bokulich et al., 2014; García-Palacios et al., 2015; Jiang et al., 2017). Thus, it is crucial to understand the impact of eCO_2_ and the presence of high-responsive rice cultivars to the belowground microbial microbiome in agroecosystems. Understanding the *modus operandi* of these systems could render farmers and policymakers to better adopt appropriate agricultural management practices to meet the future needs.

eCO_2_ often leads to soil N limitation due to the nutrients needed for stimulated plant growth (Hu et al., 2001). This situation is essential especially for the strong-responsive rice cultivars (Zhu et al., 2015). Biological nitrogen fixation, which is catalyzed by nitrogenase that is produced by broadly phylogenetic distributed but limited number of microbes, contributes 128 Tg N per year in terrestrial ecosystems (Galloway et al., 2004). Thus, investigation on soil diazotrophs is speculated to improve our understanding of the agroecosystem that operates under species-specific stimulation of crop growth via eCO_2_. During the past decades, the nitrogenase reductase subunit, encoded by *nifH* gene (Poly et al., 2001), has been widely used to study the diazotrophic communities in various ecosystem, resulting in prominent insights into terrestrial N cycling (Yeager et al., 2004; Hsu and Buckley, 2009). Thus, the evaluation of the influence of eCO_2_ on diazotrophic diversity and community structure, derived from *nifH* gene, in weak- and strong-responsive cultivars in a paddy ecosystem is of major interest. However, to our knowledge, such information is rarely available.

Recent advance of molecular technologies, such as high-throughput sequencing, provides revolutionary tools for revealing the diversity and structure of diazotrophic community, as well as how the environmental factors drive these microbes (Collavino et al., 2014); but these approaches scarcely describe the interactions between the microbial species within a community or with their environments. The co-occurrence ecological network analysis is now widely used to identify potential biotic interactions, habitat affinities, or shared physiologies from high-throughput sequencing datasets (Barberán et al., 2012; Dini-Andreote et al., 2014; Jiang et al., 2016). Based on exploring the direct or indirect interactions between microbial phylotypes, the co-occurrence network provides with another dimension in examining the relationships within the microbial community beyond the composition and diversity metrics (Barberán et al., 2012; Davison et al., 2016). For example, Tu et al. (2016) found long-term eCO_2_ did not change the *nifH* diversity and diazotrophic community structure, but it significantly changed the co-occurrence network patterns of free-living and symbiotic diazotrophs in a grassland ecosystem. Furthermore, network analysis could offer new insights into hub species and significant module associations in biotic community, and their responses to environmental conditions (Zhou et al., 2011; Deng et al., 2012). Therefore, investigating the inter-taxa associations across complex and diverse diazotrophic community may help to ascertain the diazotrophic functional roles involved in the replenishment of N in the eCO_2_-enriched ecosystem. However, there are relatively rare studies documenting the responses of soil diazotrophic microbiome to long-term eCO_2_ in agroecosystems, especially in different responsive rice cultivars.

In this study, taking advantage of the free-air CO_2_ enrichment (FACE) platform established from 2004, we aimed to verify the responses of soil diazotrophs in two rice cultivars with different performance under eCO_2_ (i.e., strong-responsive rice cultivars vs. weak-responsive rice cultivars). The following hypotheses were tested: (1) eCO_2_ could alter the abundance, the diversity, and the community structure of diazotrophic communities in strong-responsive rice cultivars to a greater extent than those of weak-responsive rice cultivars, and (2) different responses of diazotrophic co-occurrence network patterns to eCO_2_ for weak-responsive and strong-responsive cultivars. We believe that this information could provide novel insights into our understanding on the microbial ecology of the diazotrophs in response to eCO_2_, and would assist farmers and policymakers to develop strategies to breed high yield cultivars under future climate scenarios.

## Materials and Methods

### Site Description and Sample Collection

**FIGURE 1:**
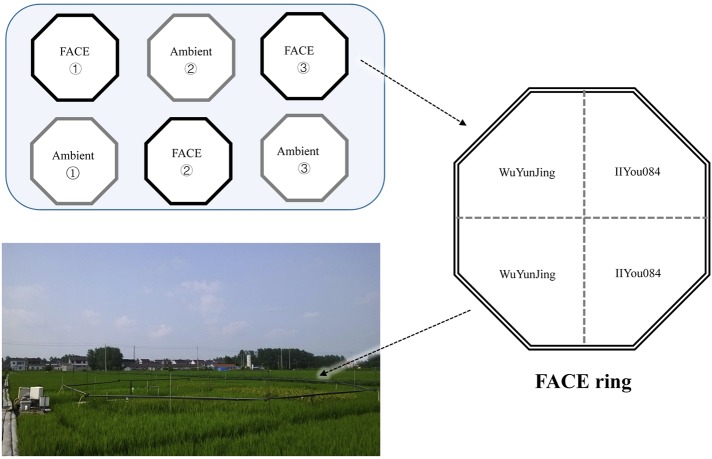
Free-air CO_2_ enrichment (FACE) system in Yangzhou City, Jiangsu, China (119°42′0′′E, 32°35′5′′N).

The study was conducted within the free-air CO_2_ enrichment (FACE) system located at Zongcun Village, Yangzhou City, Jiangsu Province, China (119°42′0′′E, 32°35′5′′N) (**Figure 1**). The long-term experimental platform was initially established in 2004, with a rice-wheat rotation crop system. From 2010, the rice-wheat rotation system was changed to a rice-fallow system. The studied soil in the region is Shajiang-Aquic Cambisol, with 9.2% sand (1–0.05 mm), 65.7% silt (0.05–0.001 mm), 25.1% clay (<0.001 mm), 1.2 g cm^-3^ bulk density, 1.5% soil organic C (SOC), 1.59 g kg^-1^ total N, 1.23 g kg^-1^ total P, 10.4 mg kg^-1^ available P and pH = 6.8 at 0–15 cm depth. The study region has a north subtropical monsoon climate with a mean annual precipitation of 900–1000 mm, and mean annual temperature of 16°C.

The FACE system has a split-plot design with CO_2_ as the main factor, and rice cultivar as the split-plot factors. More details can be found in the former research (Harvey et al., 1983; Zhu et al., 2016). Briefly, the elevated CO_2_ treatments (hereinafter referred to as eCO_2_) are operated in three octagonal rings with target CO_2_ concentration 200 ± 40 μmol mol^-1^ higher than the ambient condition. Triplicate treatments with ambient CO_2_ concentration (hereinafter referred to as aCO_2_) are conducted near the eCO_2_ rings (**Figure 1**). Each eCO_2_ ring has a diameter of 12.5 m. Towards the center of FACE rings, pure CO_2_ at high pressure was released about 50 cm above the crop canopy all day long from tubes surrounding crops. The target CO_2_ concentration within eCO_2_ rings was controlled by real-time CO_2_ monitoring system. Two rice cultivars were planted in both FACE and ambient rings (**Figure 1**). One rice cultivar is an *Indica* rice cultivar IIYou084, which showed a strong response to CO_2_ elevation (+30% yield increase). Another rice cultivar is a *Japonica* rice WuYunJing, which showed a weak response (+13% yield increase) (Zhu et al., 2015). Urea basal fertilizer was applied before rice transplanting (40% of total dose), tillering (30%), and heading (30%) stage. The P and K basal fertilizers were applied before transplanting at 9 g P_2_O_5_ m^-2^ and 9 g K_2_O m^-2^. Soil samples from two CO_2_ treatments and two rice cultivars were collected at the rice anthesis stage in 2015. To satisfy the demand of statistical and ecological network analysis, twelve parallel samples were collected for each treatment or each cultivar. For each sample, five soil cores were randomly sampled with a 3.5 cm diameter corer at 0–15 cm depth and combined into one composite sample. Totally, 48 soil samples were collected. Soil samples were stored at 4°C for chemical analysis or at -40°C for molecular analysis.

### Soil Biochemical Analysis

Dissolved organic carbon (DOC) and nitrogen (DON) was extracted from 10 g fresh soil using 50 mL ultrapure water by centrifugation at 8000 *g* for 10 min. The filtrate from a 0.45 mm filter membrane was analyzed with a total C analyzer (Elementar, Germany) and a continuous flow analyzer (Skalar, Holland), respectively. Mineral N (MN) was calculated by the sum of NH_4_^+^-N and NO_3_^-^-N, which were extracted with 2 M KCl in a 1:5 (soil:water) suspension and determined by a continuous flow analyzer (Skalar, Holland).

Soil genomic DNA was extracted from the same amount of moist soil (0.5 g) using a FastDNA^®^ SPIN Kit for soil (MP Biomedicals, Santa Ana, CA, United States). The extracted soil DNA was dissolved in 50 μl of TE buffer, quantified by a spectrophotometer (NanoDrop ND-1000, Rockwood, TN, United States) and stored at -20°C until further use. All of 48 soil samples were used for high-throughput sequencing. For each sample, the primer set, PolFI and AQER (Poly et al., 2001; Wartiainen et al., 2008), was used to amplify *nifH* gene fragments for sequencing on the Illumina Miseq sequencing platform. The oligonucleotide sequences included a 5-bp barcode fused to the forward primer as follows: barcode + forward primer. PCR was carried out in 50-μl reaction mixtures with the following components: 4 μl (initial 2.5 mM each) of deoxynucleoside triphosphates, 2 μl (initial 10 mM each) of forward and reverse primers, 2 U of Taq DNA polymerase with 0.4 μl (TaKaRa, Japan), and 1 μl of template containing approximately 50 ng of genomic community DNA as a template. Thirty-five cycles (95°C for 45 s, 56°C for 45 s, and 72°C for 60 s) were performed with a final extension at 72°C for 7 min. The purified barcoded PCR products from all of the samples were normalized in equimolar amounts, and then the library for sequencing was prepared using TruSeq^TM^ DNA Sample Prep LT Kit. A 7-nM prepared DNA library mixed with 10% PhiX control was applied for sequencing using MiSeq Reagent Kit v3 (600-cycles-PE) following the manufacturer’s protocols. Sequences obtained from this research were submitted in the NCBI Sequence Read Archive (SRA) with accession number SRP 136673.

Quantitative PCR (qPCR) of the *nifH* gene was carried out with a primer set PolFI/AQER on C1000^TM^ Thermal Cycler equipped with CFX96^TM^ Real-Time system (Bio-Rad, United States). Thermal cycler protocols and amplification conditions were referred to Watanabe et al. (2013). Briefly, The 25 μl reaction mixture contained 12.5 μl of SYBR^®^ *Premix Ex Taq*^TM^ (TaKaRa), primer set (0.5 μM each), 200 ng BSA μl^-1^, 1.0 μl template containing approximately 2–9 ng DNA. The program had an initial denaturation at 95°C for 5 min, followed by 40 cycles of denaturation at 94°C for 10 s, annealing at 55°C for 20 s and extension at 72°C for 30 s. Standard curves were obtained using 10-fold serial dilutions of the linear *Escherichia coli*-derived vector plasmid pMD18-T (TaKaRa) containing a cloned target gene, using 10^2^ to 10^8^ gene copies μl^-1^. The negative control was run with water as the template instead of soil DNA extract. The specificity of the amplification products was confirmed by melting curve analysis, and the expected sizes of the amplified fragments were checked on a 1.5% agarose gel. Each reaction was performed in triplicate and amplification efficiencies of 97.4–104% were obtained with *R^2^* values of 0.972–0.991.

### Processing of the Sequencing Data

The *nifH* gene data were processed using the Quantitative Insights Into Microbial Ecology (QIIME) 1.9.1 pipeline (Caporaso et al., 2010^1^) using default parameters unless otherwise stated. Briefly, the sequences were binned into OTUs using a 90% identity threshold, and the most abundant sequence from each OTU was selected as a representative sequence for that OTU. Taxonomy was assigned to diazotrophic OTUs against a subset of the RDP database^2^. UPARSE was used to align OTU, trim quality and remove chimera OTU (Edgar, 2013). The obtained sequences were randomly pruned to 17,000 per soil sample, since an even depth of sampling is required for α and β diversities comparison (Shaw et al., 2008). Bray–Curtis distance of taxonomic dissimilarity was calculated with *vegan* package in R and was visualized using non-metric multidimensional scaling (NMDS) plots. PERMANOVA analysis was calculated using *vegan* package in R software according to Anderson et al. (2017). The phylogenetic tree was built by using FastTree based on aligned representative sequences (Price et al., 2010).

### Co-occurrence Network Analysis

To reveal the variations in the interactions between phylotypes responding to eCO_2_ and rice cultivars, the phylogenetic molecular ecological networks (pMEN) were conducted using the random matrix theory (RMT)-based network approach (Luo et al., 2007). The pMEN construction and analyses were performed using a pipeline written in Java and Perl scripts (Deng et al., 2012)^3^. All the obtained *nifH*-OTUs were applied for the network analysis. The pair-wise correlations with the value more than 0.93 were kept, and the *p* value cutoff was set at 0.01 level. To further quantify the topology of networks, a set of indices, such as density, average centralization of degree, transitivity, average degree, average path distance and geodesic efficiency of pMEN were used to evaluate the changes of biotic interactions within the diazotrophic communities in response to eCO_2_ and rice cultivars. The networks were visualized using the interactive platform *Gephi* (Bastian et al., 2009).

### Statistical Analysis

Statistical procedures were calculated using the IBM Statistical Product and Service Solutions (SPSS) (Version 23). The data were expressed as means with standard deviation (SD). The asterisk after numbers indicates the significant difference between eCO_2_ and aCO_2_ for the same rice cultivar using the Student’s *t*-test. Differences with a *p* < 0.05 were considered statistically significant. Mantel test was applied to detect the partial relationships with *vegan* package in R (999 permutations). White’s non-parametric *t*-test and related plots were performed in STAMP v2.1.3 (Parks et al., 2014).

## Results

### The α-Diversity Indices and Abundance of Soil Diazotrophic Communities

Total 833,149 reads targeting *nifH* gene were obtained from 48 soil samples after quality trimming, frameshift correction and chimera removal. A random re-sampling effort of 17,000 reads per soil sample was made for the downstream statistical analyses. Generally, these reads were clustered into 3,005 *nifH* OTUs. Up to 90.6% of these OTUs were affiliated with Proteobacteria phylum (Supplementary Figure S1). The rest was classified to Euryachaeota (5.3%), Firmicutes (1.9%), and Bacteroidetes (1.6%). On the order level, diazotrophic OTUs were affiliated with 13 microbial orders. Amongst, the top five microbial orders were *Rhizobiales* (32.13%), *Rhodocyclales* (21.80%), *Burkholderiales* (13.32%), *Syntrophobacterales* (6.16%), and *Methanosarcinales* (5.10%).

Based on the obtained *nifH* OTUs, four α-diversity indices, including Shannon index, estimated richness (Chao1), OTU richness (Observed OTUs) and phylogenetic diversity (PD index) were calculated and compared among different treatments (**Table 1**). Statistical analyses showed that eCO_2_ could not significantly affect all four α-diversity indices in the weak-responsive cultivar soils (*p* > 0.05), while for the strong-responsive cultivars the diazotrophic α-diversities significantly decreased under eCO_2_ in comparison with those of aCO_2_ (*p* < 0.05).

**Table 1:** The effects of CO_2_ (ambient [aCO_2_] and elevated [eCO_2_]) and cultivar (weak- and strong-responsive) on the α-diversity indices of diazotrophic communities and the *nif*H gene copy numbers (10^7^/g dw soil).

	Weak-responsive cultivar		Strong-responsive cultivar	
	aCO_2_	eCO_2_	aCO_2_	eCO_2_
Shannon Index	8.35 ± 0.15	8.36 ± 0.13	8.13 ± 0.09	7.85 ± 0.45*
Observed_OTU	1954 ± 73	1960 ± 43	1908 ± 68	1798 ± 160*
Chao1 Index	2532 ± 90	2543 ± 91	2526 ± 88	2390 ± 175*
Phylogenetic diversity	117.4 ± 6.43	117.6 ± 2.73	114.8 ± 5.68	107.8 ± 8.00*
*nif*H gene copies	5.73 ± 0.32	6.47 ± 0.29*	6.57 ± 0.48	7.83 ± 0.59*

*Values are means ± S.D. (*n* = 12). The asterisk (^∗^) after the numbers means significant difference between aCO_*2*_ and eCO_*2*_ treatments for the same rice cultivar (Student’s *t*-test, *p* < 0.05)*.

The abundance of *nifH* genes was estimated using quantitative PCR (qPCR). The *nifH* gene copy number differed under different treatments, ranging from 5.73 × 10^7^ to 7.83 × 10^7^ (per g d.w.s) (**Table 1**). Overall, the *nifH* gene abundance increased by 12% ∼ 19% under eCO_2_ than under aCO_2_. Student’s *t*-test showed that eCO_2_ significantly increased the *nifH* gene abundance compared to that of aCO_2_ (*p* < 0.05) for both of the rice cultivars. The strong-responsive cultivar increased *nifH* gene copies to a greater extent under eCO_2_ than the weak-responsive one did under the same condition.

### The Composition of Diazotrophic Community

**FIGURE 2:**
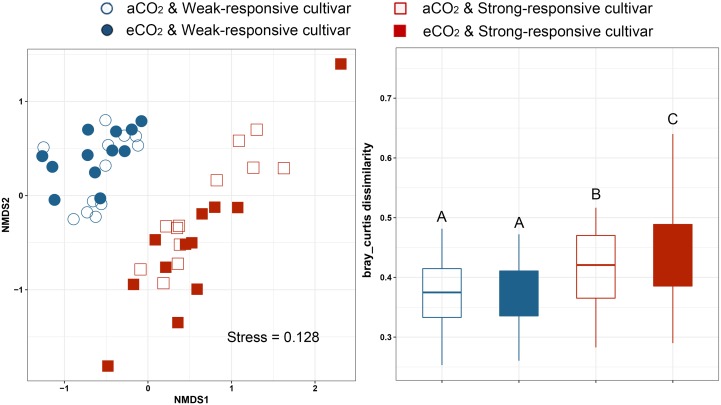
Non-metric multidimensional analysis of community dissimilarities within different groups basing on Bray-Curtis distance. **(A)** NMDS plot; **(B)** box plots based on dissimilarity of diazotrophic communities under different treatments.

Non-metric multidimensional scaling analyses (NMDS) based on Bray–Curtis distance was conducted to assess the dissimilarities of the diazotrophic communities among different treatments (**Figure 2**). It is clearly shown that the communities were clustered between the two different responsive rice cultivars (**Figure 2A**). The dissimilarity of Bray–Curtis distance between aCO_2_ and eCO_2_ was not significant for the weak-responsive cultivars, while significant dissimilarity between eCO_2_ and aCO_2_ was observed for strong-responsive cultivars (**Figure 2B**). The above findings are confirmed by PERMANOVA analysis (Supplementary Table S1). Mantel test was conducted to reveal the correlations between diazotrophic community composition and soil properties, i.e., SOC, MN, and DON (Supplementary Table S2). With regards to the dissimilarity of diazotrophic community, it was significantly correlated with SOC (*r* = 0.19, *p* = 0.009) and MN (*r* = 0.14, *p* = 0.030) and DON (*r* = 0.18, *p* = 0.019) (Supplementary Table S2).

### The Significantly Responded Diazotrophic Phylotypes

**FIGURE 3:**
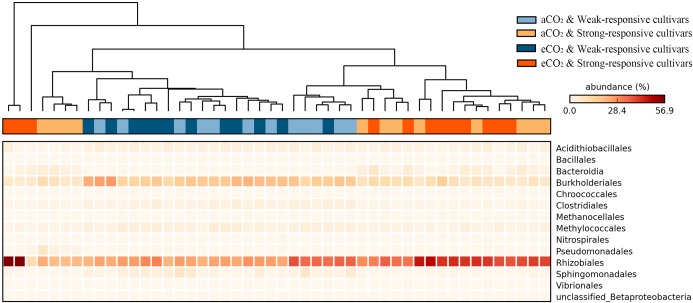
Heatmap of the significant responded diazotrophs at order level in paddy soil with different treatments (Tukey–Kramer test, *p* < 0.05). The color intensity in each panel shows the relative abundance (percentage) of each phylotype at order level in each sample.

The significance of the shifts of the diazotrophic phylotypes between different treatments was statistically evaluated at the STAMP platform. The heatmap shows a data matrix where color code gives an overview of the numeric differences at order level (**Figure 3**). The detailed significant differences at genera level were checked by using White’s non-parametric *t*-test (Supplementary Figure S2). Overall, there were 14 diazotrophic orders significantly changed between different treatments (*p* < 0.05). The *Rhizobiales* order and the *Candidatus* *Azobacteroides*, *Mesorhizobium*, and *Methylobacterium* genera, the typical symbiotic diazotrophs (Yanni et al., 1997; Shu et al., 2012), were significantly increased for the strong-responsive cultivars (39.5% in total) in comparison to weak-responsive ones (28.0% in total) (**Figure 3** and Supplementary Figure S2). On the contrary, the free-living diazotrophs, such as *Burkholderriales* and *Clostridiales* (Kimble and Madigan, 1992; Brook et al., 1997), were more predominant for the weak-responsive cultivars (19.7% in total) rather than for the strong-responsive ones (8.9% in total) (**Figure 3** and Supplementary Figure S2). The significant responses of diazotrophic phylotypes to eCO_2_ were verified by White’s non-parametric *t*-test (Supplementary Figure S2). In response to eCO_2_, the weak-responsive cultivars increased *Spirochaeta*, *Desulfobulbaceae,* and *Syntrophobotulus* by 13.6, 2.4, and 1.7 times specifically, while the strong-responsive cultivars decreased *Azospira*, *Thiorhodospira*, *Accumulibacter,* and *Cupriavidus* by 1.97% in total.

### Co-occurrence Network Patterns of Soil Diazotrophs

**FIGURE 4:**
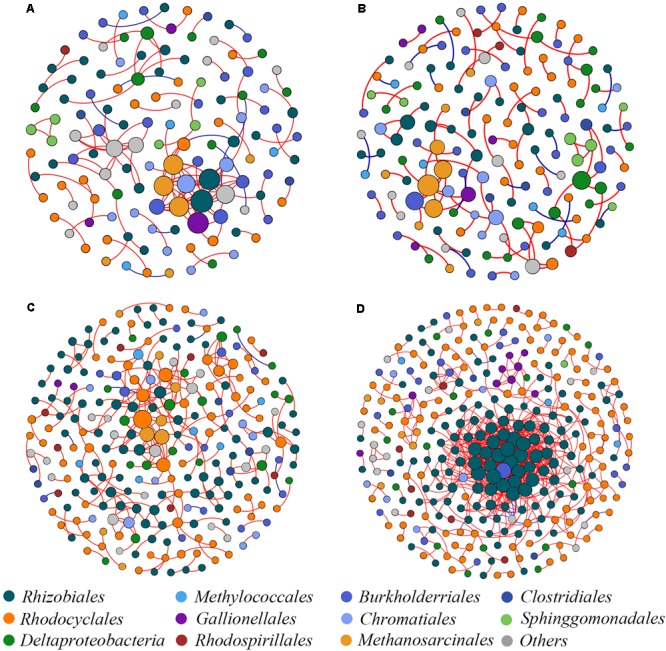
Network co-occurrence analysis of diazotrophic communities basing on *nifH* gene. **(A)** aCO_2_ & weak-responsive cultivar; **(B)** eCO_2_ & weak-responsive cultivar; **(C)** aCO_2_ & strong-responsive cultivar; **(D)** eCO_2_ & strong-responsive cultivar. Each dot represents a *nifH*-OTU (node). The size of each node is proportional to number of connections. Each node is labeled at order level.

Four co-occurrence ecological networks of soil diazotrophs were generated from different CO_2_ concentration treatments and from different responsive rice cultivar soils. (**Figure 4**). The value of modularity index of all networks was greater than 0.4 (**Table 2**), suggesting that all the networks had a modular structure (Newman, 2006; Barberán et al., 2012). The eCO_2_ treatments had more nodes (number of OTUs with significant correlations) than aCO_2_ treatments, regardless of weak and strong responsive cultivars. The difference of edges (number of strong and significant correlations between nodes) between eCO_2_ and aCO_2_ were inconsistent for the two different cultivars. Specifically, under eCO_2_ the number of edges decreased for weak-responsive cultivars, but increased for strong-responsive cultivars. Further analyses by using pMENs (Deng et al., 2012) showed that the topological properties of networks characterizing the complexity of inter-relationships among ecotypes were also different between eCO_2_ and aCO_2_ for both rice cultivars. Random networks were generated to test the statistical significance of the network indices between different treatments (Supplementary Table S3). Permutation tests indicated that the majority of the network indices were significantly different between eCO_2_ and aCO_2_ for the two rice cultivars. (*p* < 0.001) (Supplementary Table S4). In detail, the values of density, transitivity, average degree and average clustering coefficient were significantly lower under eCO_2_ than aCO_2_ for the weak-responsive cultivars, while these values were significantly higher under eCO_2_ for the strong-responsive cultivars (*p* < 0.05) (**Table 2**). When the surround conditions changed from aCO_2_ to eCO_2_, the hub members with maximum stress centrality in the weak-responsive cultivar treatments shifted from *Halorhodospira* to *Sideroxydans*, while the hub diazotroph in the strong-responsive cultivar treatments shifted from *Bradyrhizobium* to *Dechloromonas*.

**Table 2:** Topological properties of networks obtained within each group of treatments.

Network metrics	Weak-responsive cultivar		Strong-responsive cultivar	
	aCO_2_	eCO_2_	aCO_2_	eCO_2_
Modularity	0.792	0.959	0.931	0.464
Number of nodes	131	157	250	317
Total number of edges	128	103	235	829
Number of positive correlations	118	87	228	817
Number of negative correlations	10	16	7	12
Density	0.015	0.0080	0.008	0.017
Transitivity	0.585	0.417	0.439	0.562
Centralization of degree	0.071	0.024	0.045	0.108
Average degree	1.954	1.312	1.88	5.23
Maximal degree	11	5	13	39
Average clustering coefficient	0.152	0.082	0.181	0.259
Centralization of betweenness	0.006	0.002	0.003	0.012
Connectedness	0.042	0.014	0.023	0.141
Efficiency	0.726	0.557	0.764	0.899
Geodesic efficiency	0.601	0.752	0.594	0.422
Harmonic geodesic distance	1.664	1.329	1.684	2.37
Centralization of eigenvector centrality	0.344	0.491	0.435	0.199
Maximal eigenvector centrality	0.37	0.505	0.448	0.22
R square of power-law	0.834	0.973	0.925	0.797
Nodes with max stress centrality	*Halorhodospira*	*Sideroxydans*	*Bradyrhizobium*	*Dechloromonas*

## Discussion

At elevated CO_2_, low supply of N in the soil could limit the capacity of plants to fix CO_2_ photosynthetically and potentially restrict the increases in plant growth and yield over time through long-term C and N dynamics (Rastetter et al., 1997; McMurtrie et al., 2000). Strong-responsive crop cultivars could extremely increase the soil N uptake under eCO_2_ (Zhu et al., 2015). Information on the N cycling related diazotrophic microbiome could enrich the knowledge on the sustainability of ecosystem when building up high-yielding agroecosystem to adapt the future climate scenarios (Reich et al., 2006).

### Responses of Diazotrophic Abundance to eCO_2_ and Rice Cultivars

In this study, eCO_2_ significantly increased the soil diazotrophic abundance for both weak- and strong-responsive cultivars. This result is generally in keeping with previous studies (Xu et al., 2013; Tu et al., 2017), in which increased *nifH* gene abundance was observed under eCO_2_ in a grassland ecosystem based on the shotgun metagenome sequencing and GeoChip technology. Lee et al. (2017) also demonstrated that the microbial abundance was sensitive to eCO_2_ in a salt marsh ecosystem. eCO_2_ often lead to soil N limitation due to the increased nutrients need for stimulated plant growth (Luo et al., 2004; Tu et al., 2016). The increase of diazotrophs could partly tackle the shortage of available N in soil by converting more atmospheric N into ammonia (Giller and Cadisch, 1995). Thus, increased N limitation might promote the increase of belowground diazotrophs under eCO_2_. Additionally, the increased DOC could provide with adequate carbon source for soil microorganisms under eCO_2_ conditions. The stimulated C allocation via root biomass (Hu et al., 2017) and plant exudates (Zhu et al., 2016) under eCO_2_ could provide with more energy derived from C sources for soil microbial growth (Drigo et al., 2008; Blankinship et al., 2011). Our previous studies carried out on the same FACE platform demonstrated that high-yielding rice cultivars increase soil N uptake under eCO_2_ (Zhu et al., 2015). Thus, higher need of available N in soil stimulated higher increase of the diazotrophic abundance for the strong-responsive cultivars in comparison to that of the weak-responsive ones under eCO_2_. These results indicate that soil diazotrophs can increase their abundance to regulate available N according to crop growth and soil N limitation under eCO_2_ for different responsive cultivars.

### Responses of Diazotrophic Diversity to eCO_2_ and Rice Cultivars

The diazotrophic diversity was not changed under eCO_2_ for weak-responsive cultivars, while it significantly decreased for strong-responsive cultivars under eCO_2_. This result affirmed our first hypothesis that eCO_2_ could alter the diazotrophic community in strong-responsive rice cultivars to greater extent than that of weak-responsive rice cultivars. Although low N supply under eCO_2_ can stimulate the diazotrophic abundance, some diazotrophic species were probably not well adapted to the changed biochemical properties of strong-responsive cultivar treatments, such as increased dissolved organic C (Hu et al., 2017), stimulated root turnover (Zhu et al., 2016), and altered soil protozoa and nematodes under eCO_2_ (Neher et al., 2004; Mueller et al., 2016; Hu et al., 2017). For example, in this study, the free-living diazotrophs, such as *Azospira*, *Thiorhodospira*, *Accumulibacter,* and *Cupriavidus* genera, were significantly decreased for strong-responsive cultivars in response to eCO_2_. It is also found that the symbiotic diazotrophs were positively increased in the strong-responsive cultivar soils. Soil microbial responses are the indirect result of faster root growth and increased rhizodeposition in response to eCO_2_ (Kandeler et al., 1998). Thus, higher stimulation of root biomass in strong-responsive cultivar treatments (Zhu et al., 2016) could influence the distribution of the symbiotic and free-living diazotrophs via physical isolation by the crop roots. Meanwhile, the higher extent of increased N uptake from strong-responsive cultivar soils could increase the competition between different microbial phylotypes (Hu et al., 2001; Tu et al., 2016). Consequently, the diversities significantly decreased for strong-responsive cultivars under eCO_2_. Additionally, the diazotrophic dissimilarity was found significantly correlated with mineral N and dissolved organic N in this study. It is indicated that soil diazotrophs could change their composition to regulate soil N availability according to the surround situation of N limitation resulted from eCO_2_ for different cultivars. This regulating ability of soil diazotrophs on soil N limitation may potentially alter the sustainability of soil ecosystem (Reich et al., 2006).

### Divergent Interactions of Diazotrophic Phylotypes in Weak- and Strong-Responsive Rice Cultivars Under eCO_2_

Network analyses could provide more detailed information about the interactions between the microbial phylotypes compared to the standard alpha/beta diversity metrics that are usually used in microbial ecology (Barberán et al., 2012; Davison et al., 2016). Tu et al. (2016) employed a random matrix theory (RMT)-based network approach to discern phylogenetic diazotrophic ecological networks using metagenomic sequencing in a grassland ecosystem. They found the shifted structure of the identified networks under aCO_2_ and eCO_2_ conditions, mainly in terms of the network composition, the role of individual diazotrophic phylotypes and the interactions between diazotrophic species. In this study, network analysis by pMEN analysis was applied to generate the co-occurrence networks of the diazotrophic microbiome. Such a rational analysis provided us with an opportunity to identify potential diazotrophic interactions between different treatments, as well as to observe how the microbial patterns affect the ecosystems properties (Zhou et al., 2011; Dini-Andreote et al., 2014).

In this study, divergent responses of diazotrophic co-occurrence network were observed between weak- and strong-responsive cultivars in response to eCO_2_. These results confirmed our second hypothesis. In the network analyses, edges, which connects two nodes, represents the strong and significant correlations between two functional species (nodes) (Newman, 2006). More edges suggest higher-level species-species interactions within the ecological network (Newman, 2006). This study found that eCO_2_ led to more nodes than that of aCO_2_ for both cultivars. The number of edges was significantly increased for strong-responsive cultivars but decreased for weak-responsive cultivars in response to eCO_2_. These phenomena indicated the distinct responses of soil diazotrophic interactions to different rice cultivars under eCO_2_. Further pairwise comparison revealed that larger average harmonic geodesic distances and the smaller geodesic efficiencies were observed for strong-responsive cultivars under eCO_2_. According to the streamlining theory, the increased competition for resources could promote the interactions between different microbial members (Giovannoni et al., 2014). Consistently, it is demonstrated that microorganisms tend to coexist for survival when the surrounding energy resources are limited based on the first principles of thermodynamics models (Grosskopf and Soyer, 2016). Thus, both the eCO_2_ and the strong-responsive cultivars promoted the diazotrophic interspecies dependency. This is exemplified by the fact that the degrees of centralization, complexity and transitivity increased under eCO_2_ than under aCO_2_ for strong-responsive cultivars, while these properties showed an opposite trend in response to eCO_2_ for weak-responsive cultivars. In network analysis, degrees of centralization and complexity represents the ability of inter-species network to avoid cascading collapses when it undergoes environmental disturbances (Deng et al., 2012). Low degrees of centralization and complexity is of great help towards the stability of ecosystem (Liang et al., 2016). The transitivity in network topology is an indicator that the network structure is dominated by hub species-species interactions and that their absence could have a disproportionate effect on the overall community structure (Berry and Widder, 2014). Lower transitivity suggests weaker interactions and couplings within the community (Narisawa et al., 2008) but stronger stability of the ecological network (Wood et al., 2017). Hence, the networks of soil diazotrophs were more stable under aCO_2_ than under eCO_2_. At eCO_2_, strong-responsive cultivars destabilized the diazotrophic network, while weak-responsive cultivars developed a more stable diazotrophic network. As discussed above, the differences of soil N uptake and belowground C allocation might be the reasons for the divergent responses of diazotrophic networks to eCO_2_ between weak- and strong-responsive cultivars. Reich et al. (2006) pointed out that the sustainability of an ecosystem could be constrained by the N limitation under eCO_2_. Our study further demonstrated that strong-responsive cultivars could diminish the stability of the diazotrophic ecological network and potentially influence the sustainability of the agroecosystem in response to CO_2_.

### Most Distinctly Responded Hub Diazotrophs in Ecological Networks

Hub phylotypes are generally crucial to the entire network, and their absence may cause catastrophic changes in the ecosystem (Dunne et al., 2002). In this study, it is found that the node with the maximum stress centrality shifted from *Halorhodospira* under aCO_2_ to *Sideroxydans* under eCO_2_ for weak-responsive cultivars, and shifted from *Bradyrhizobium* under aCO_2_ to *Dechloromonas* under eCO_2_ for strong-responsive cultivars. It is well known that the Fe protein in nitrogenase is a reductase which has a high reducing power and is responsible for the supply of electrons (Burgess and Lowe, 1996). Moreover, *Sideroxydan*, which has a high similarity sequence with *nifH* gene, is described as Fe(II)-oxidizing bacteria (Soni et al., 2016). The metabolism of nitrogenase could directly utilize Fe ion within the cell of *Sideroxydan* genus. Thus, it is reasonable that *Sideroxydans* genus became one of the hub diazotrophs in the network of weak-responsive cultivar soils under eCO_2_. For strong-responsive cultivars, *Dechloromonas* genus was the hub diazotrophs in the network under eCO_2_. Similar with the *Sideroxydans* genus in weak-responsive cultivar soils, the capabilities on oxidation of both aqueous and chelated Fe(II) (Chakraborty and Picardal, 2013) could help *Dechloromonas* become the hub species in strong-responsive cultivar soils. It is also demonstrated that *Dechloromonas* genus has a broad range of novel metabolic capabilities and bioremediative applicability on degrading several kinds of organic compounds in soil with complex life-style (Salinero et al., 2009), which might potentially provide with energy resources for the other soil diazotrophs. It is speculated that when the hub species shifted to *Dechloromonas* in the strong-responsive cultivar soils, the pivots of dependency in the diazotrophic network could be deepened under eCO_2_. This, combined with the responses of topological properties, implies that the shifting of hub soil diazotrophic species from *Bradyrhizobium* to *Dechloromonas* makes the ecosystem unstable under eCO_2_ for strong-responsive cultivars. Surely, these microbial mechanisms need to be further explored and verified by more pure culture experiments.

## Conclusion

Elevated atmospheric CO_2_ can stimulate plant growth but often leads to N limitation in soil. Understanding the impacts of eCO_2_ on the soil diazotrophic microbiome in different responded rice cultivars can provide with comprehensive insights into the response and stability of the soil microbial ecosystem under future climate scenarios. Our results showed divergent responses of the diazotrophic microbiome to the two different rice cultivars with distinctly weak and strong responses to eCO_2_. The diazotrophic abundance significantly increased for both rice cultivars to satisfy the increased N demand under eCO_2_. eCO_2_ caused a decrease in the taxa diversity and separated the dissimilarity of diazotrophic communities in the strong-responsive cultivars, but it did not change the diazotrophic composition for the weak-responsive ones. Network analysis revealed that when the surround conditions switched from aCO_2_ to eCO_2_, the strong-responsive cultivars destabilized the diazotrophic community by complicating and centralizing the co-occurrence network, while the network pattern was simplified and decentralized in weak-responsive cultivar soils. These divergent responses may promote further understanding of the strategies and the stability of soil microbial communities when breeding high-yielding cultivars under eCO_2_. However, *in situ* studies coupled stable isotope probing and the co-occurrence network analysis at species level should be further considered to detect actively interacting microbes under prospective climatic scenarios (Ho et al., 2016).

## Author Contributions

JGZ, XL, and YF designed the study. YY, CZ, and JGZ performed the experiments. YY, JWZ, and EP analyzed the data. YY, EP, MB, and YF wrote the paper.

## Conflict of Interest Statement

The authors declare that the research was conducted in the absence of any commercial or financial relationships that could be construed as a potential conflict of interest. The reviewer AH and handling Editor declared their shared affiliation.

## Footnotes

1 http://www.qiime.org

2 http://rdp.cme.msu.edu/

3 http://129.15.40.240/mena/login.cgi
