# Supplementary material for: Divergent Responses of the Diazotrophic Microbiome to Elevated CO2 in Two Rice Cultivars
Source: Front Microbiol. 2018 Jun 1;9:1139. doi: 10.3389/fmicb.2018.01139 (PMC5992744; doi:10.3389/fmicb.2018.01139)
Supplement: Supplementary file 1 [file Table_1.PDF]

Table S1 The effects of CO_2 (ambient [CO_2] and elevated [CO_2]), rice cultivars (weak- and strong-responsive cultivar) and the interactions on the diazotrophic community composition by using PERMANOVA analysis

	F value	*p*
CO_2	0.89	0.35
Cultivar	7.14	0.01
CO_2 × Cultivar	1.27	0.04

Table S2 The mean content (mg/kg) of soil resources and their correlations with diazotrophic community structure (Bray-Curtis distance) determined by Mantel test.

	Weak-responsive cultivar		Strong-responsive cultivar		$r_M(p)$
	aCO_2	eCO_2	aCO_2	eCO_2	
Soil organic C	48.7±10.41	69.1±12.6*	66.1±4.94	81.6±6.70*	0.19(0.009)
Mineral N	6.48±0.76	6.89±0.72*	7.34±0.57*	6.51±0.89*	0.14(0.030)
Dissolved organic N	4.84±0.21	4.59±0.62	6.66±0.79	4.14±0.58*	0.18(0.019)

Values are means ± S.D. (n=12). The asterisk (*) after the numbers means significant difference between aCO_2 and eCO_2 treatments for the same rice cultivar (Student's *t*-test, $p < 0.05$).

Table S3 Topological properties of the empirical molecular ecological networks (MENs) of additional microbial communities and their associated random MENs

Network Indexes	aCO_2 & Weak		eCO_2 & Weak		aCO_2 & Strong		eCO_2 & Strong	
	Empirical Network Indexes	Random Network Indexes	Empirical Network Indexes	Random Network Indexes	Empirical Network Indexes	Empirical Network Indexes	Empirical Network Indexes	Empirical Network Indexes
Average clustering coefficient	0.152	0.015±0.008	0.082	0.009±0.008	0.181	0.005±0.004	0.259	0.104±0.008
Average path distance	2.281	3.915±0.176	1.753	2.610±0.571	2.191	5.910±0.376	3.133	3.255±0.039
Geodesic efficiency	0.601	0.305±0.012	0.752	0.566±0.073	0.594	0.209±0.011	0.422	0.339±0.003
Harmonic geodesic distance	1.664	3.286±0.125	1.329	1.797±0.246	1.684	4.793±0.250	2.370	2.950±0.029
Centralization of degree	0.071	0.071±0.000	0.024	0.024±0.000	0.045	0.045±0.000	0.108	0.108±0.000
Centralization of betweenness	0.006	0.122±0.028	0.002	0.007±0.004	0.003	0.162±0.040	0.012	0.076±0.008
Centralization of stress centrality	0.018	0.223±0.052	0.002	0.007±0.005	0.005	0.260±0.075	0.085	0.394±0.043
Centralization of eigenvector centrality	0.344	0.335±0.029	0.491	0.501±0.043	0.435	0.483±0.042	0.199	0.196±0.007
Density	0.015	0.015±0.000	0.008	0.008±0.000	0.008	0.008±0.000	0.017	0.017±0.000
Transitivity	0.585	0.059±0.020	0.417	0.006±0.019	0.439	0.013±0.009	0.562	0.203±0.009
Connectedness	0.042	0.406±0.048	0.014	0.026±0.006	0.023	0.432±0.049	0.141	0.851±0.037
Efficiency	0.726	0.978±0.003	0.557	0.794±0.051	0.764	0.990±0.001	0.899	0.984±0.001

Table S4 T-test about the significance of differences of topological index between aCO_2 and eCO_2 at two different responsive cultivars

t-test	Weak-responsive cultivar	Strong-responsive cultivar
Average clustering coefficient	0.0001	0.0001
Average path distance	0.0001	0.0001
Geodesic efficiency	0.0001	0.0001
Harmonic geodesic distance	0.0001	0.0001
Centralization of degree	/	/
Centralization of betweenness	0.0001	0.0001
Centralization of stress centrality	0.0001	0.0001
Centralization of eigenvector centrality	0.0001	0.0001
Density	/	/
Transitivity	0.0001	0.0001
Connectedness	0.0001	0.0001
Efficiency	0.0001	0.0001
Modularity	0.0001	0.0001
