# Supplementary material for: Divergent Responses of the Diazotrophic Microbiome to Elevated CO2 in Two Rice Cultivars
Source: Front Microbiol. 2018 Jun 1;9:1139. doi: 10.3389/fmicb.2018.01139 (PMC5992744; doi:10.3389/fmicb.2018.01139)
Supplement: Supplementary file 2 [file Image_1.PDF]

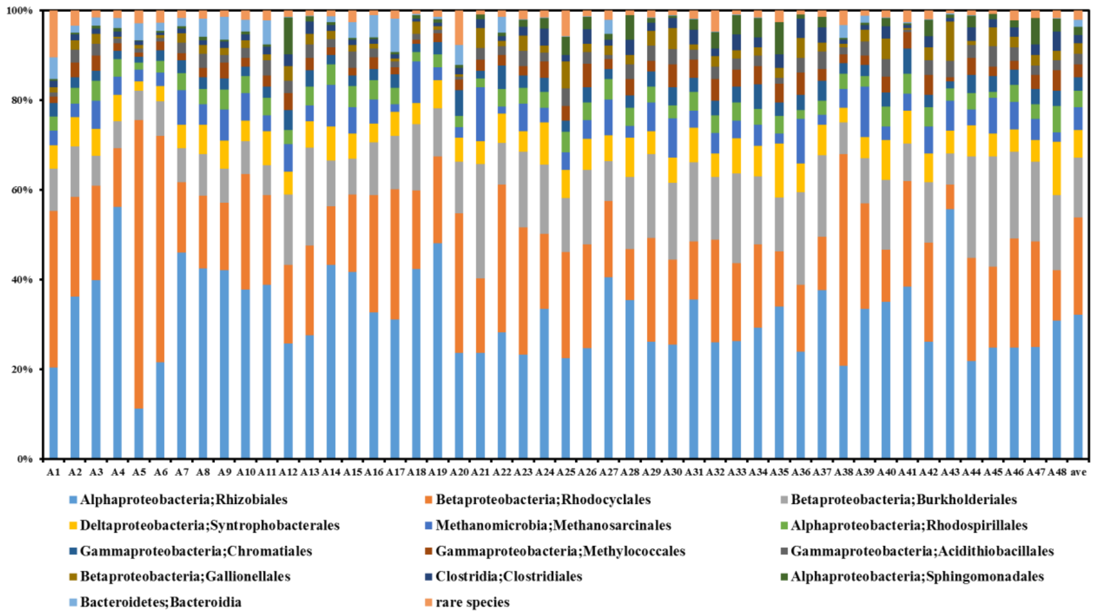

Fig. S1 The 100 % stacked column chart of relative abundances of diazotrophs at order level in each treatment.

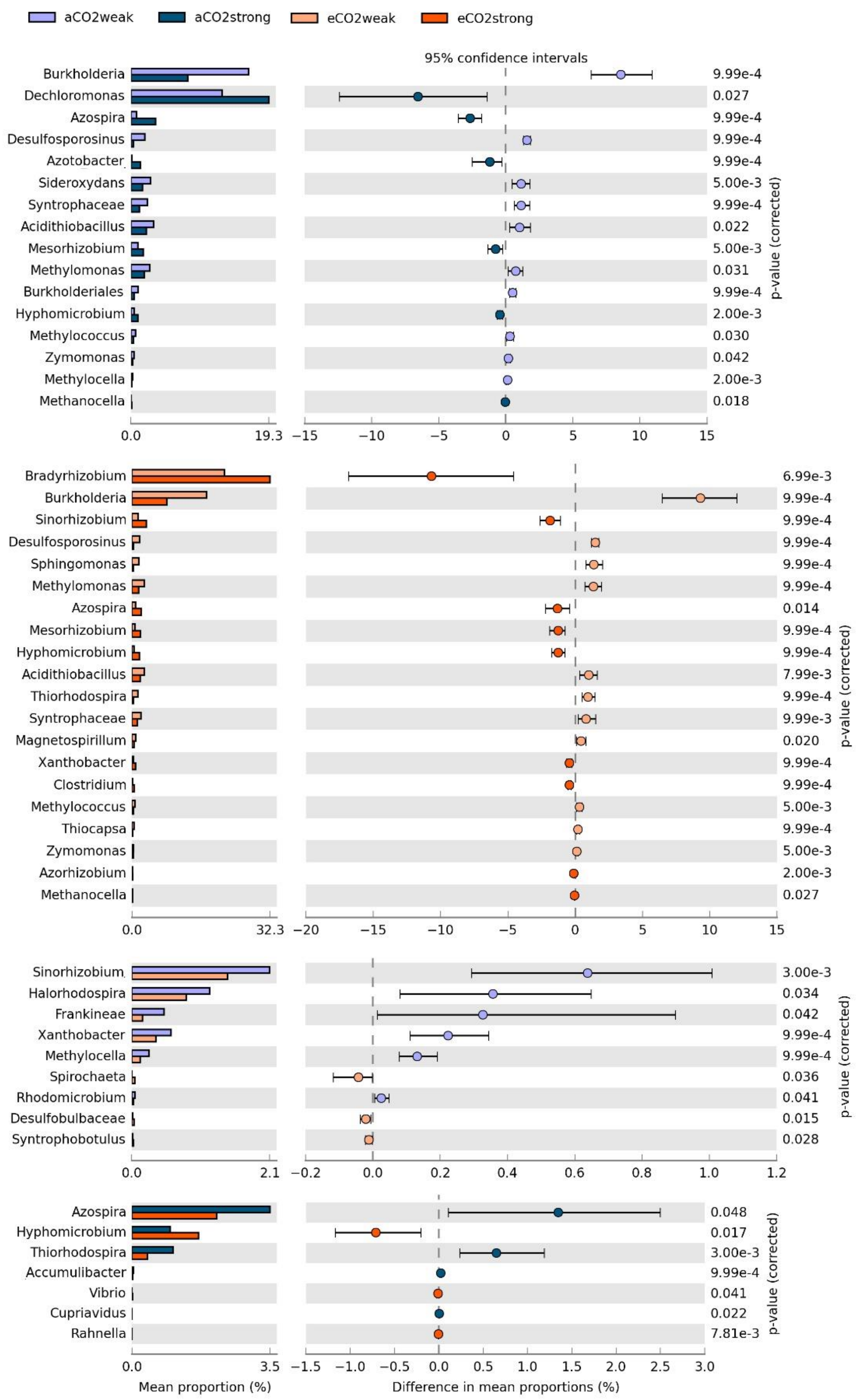

Fig. S2 Extended error bar plot considering significant changed diazotrophic genera between different treatments (p <0.05).
